# Adult Female with a Headache

**DOI:** 10.5811/cpcem.2017.1.33237

**Published:** 2017-03-15

**Authors:** Kelley A. Wittbold, Jacqueline Boehme, Heidi H. Kimberly, Sarah E. Frasure

**Affiliations:** * Brigham and Women’s Hospital, Department of Emergency Medicine, Boston, Massachusetts; † Massachusetts General Hospital, Department of Medicine, Boston, MA

## CASE REPORT

**Image 1:**
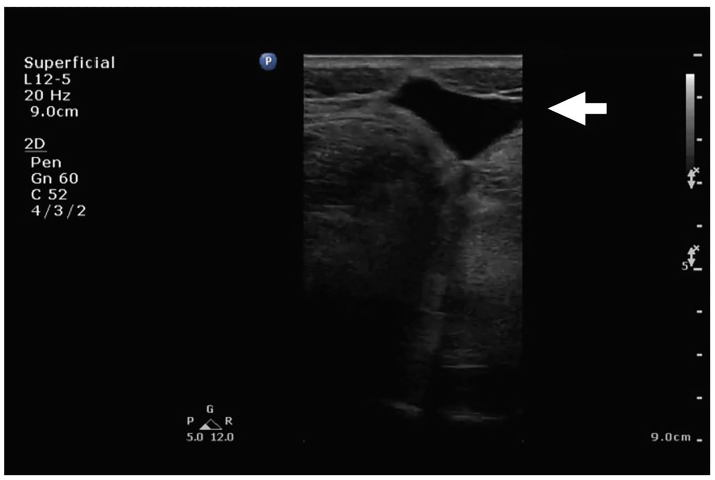
Sagittal ultrasound image of a simple fluid collection (arrow) tracking from the skin to the spine.

**Image 2:**
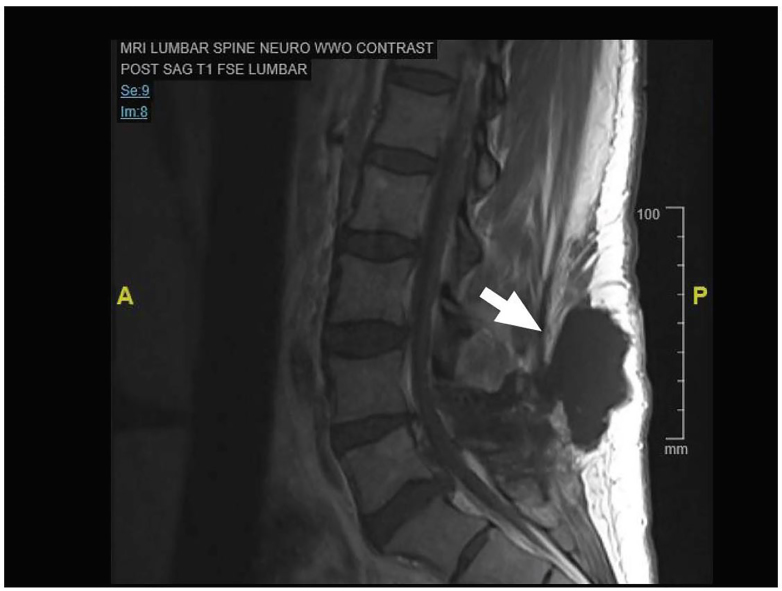
Sagittal T1-weighted magnetic resonance imaging of the lumbosacral spine demonstrating the pseudomeningocele (arrow) identified initially on ultrasound.

A 38 year old female with a history of a right foot drop after medial facetectomies (L4-L5, L5-S1) and micro-discectomy (L4-L5) eight weeks prior presented to the emergency department (ED) with two weeks of headache and neck pain. She denied fever or chills. In the ED, her vital signs were stable and her physical exam demonstrated an area of fluctuance along a well-healed surgical incision at L4-S1 that was most prominent when she sat upright. There was no overlying erythema or tenderness to palpation. The emergency physician ordered blood work, pain medication, and performed a point-of-care ultrasound along the area of fluctuance with a linear transducer (5–12 MHz). The ultrasound images demonstrated a large, hypoechoic fluid collection that tracked to the spine (Image 1, Video). A magnetic resonance image (MRI) of the lumbosacral spine confirmed the suspected diagnosis (Image 2).

## DISCUSSION

### Pseudomeningocele

The patient was taken to the operating room where a defect in the dura was identified. A pseudomeningocele is an abnormal cerebrospinal fluid collection that communicates with the dura mater surrounding the brain or spinal cord. The symptoms of pseudomeningocele include back pain, neck pain, and headache. The sensation of headache is rarely due to injury to the brain parenchyma itself, but rather due to tension, traction, dilation, or inflammation of pain-sensitive structures such as blood vessels or the dura mater.^1^ Patients with pseudomeningocele may also present with signs and symptoms of acute or chronic meningitis.^2^ MRI is considered the imaging modality of choice in the assessment of a pseudomeningocele. In this case, the clinician used point-of-care ultrasound to identify a simple fluid collection at the surgical site, which tracked from the soft tissue to the level of the dura. There was no evidence of soft tissue cobblestoning, loculations, or wall thickening of the collection to suggest cellulitis or abscess. Furthermore, there was no hyperechoic debris that is generally found inside an abscess on ultrasound imaging. MRI confirmed the suspected diagnosis.

## 

Video This sagittal ultrasound video clip, performed by the emergency physician with a linear transducer (5–12 MHz), demonstrates a simple fluid collection without overlying cobblestoning or wall thickening.

## References

[1] Adams JG, Barton ED, Collings JL (2013). Emergency Medicine: Clinical Essentials.

[2] Gass H, Goldstein AS, Ruskin R (1971). Chronic postmyelogram headache. Isotopic demonstration of dural leak and surgical cure. Arch Neurol.

